# Impact of Lockdown Measures and Meteorological Parameters on the COVID-19 Incidence and Mortality Rate in Bangladesh

**DOI:** 10.1097/IM9.0000000000000052

**Published:** 2021-02-05

**Authors:** Md. Ashik Imran, Imad Uddin Noor, Ajit Ghosh

**Affiliations:** Department of Biochemistry and Molecular Biology, Shahjalal University of Science and Technology, Sylhet-3114, Bangladesh.

**Keywords:** Bangladesh, case fatality rate, COVID-19, lockdown, meteorological parameters, non-pharmaceutical intervention

## Abstract

The coronavirus disease 2019 (COVID-19) pandemic has become a public health crisis and a global catastrophe for human societies. In the absence of a vaccine, non-pharmaceutical interventions have been implemented across the world to reduce COVID-19 transmission. Recently, several studies have articulated the influence of meteorological parameters on COVID-19 infections in several countries. The purpose of this study was to investigate the effect of lockdown measures and meteorological parameters on COVID-19 daily confirmed cases and deaths in Bangladesh. Different parameters, such as case fatality rate, recovery rate, number of polymerase chain reaction tests, and percentages of confirmed cases were calculated for data covering March to September 2020. The meteorological data include daily average temperature, humidity, and wind speed, and their effects on COVID-19 data were analyzed after 0, 3, 7, and 14 days. A linear regression analysis revealed that all the studied meteorological parameters were positively correlated with the daily new cases and deaths in Bangladesh, while the highest correlations were observed for the 14 days incubation period. These results provide useful implications for the healthcare authorities to contain the pandemic in Bangladesh and beyond.

## Introduction

Coronavirus disease 2019 (COVID-19) is a pathogenic viral infection caused by a new coronavirus strain named severe acute respiratory syndrome coronavirus 2 (SARS-CoV-2) that was first reported in Wuhan, China, in late December 2019.^1^ This disease has rapidly spread to other parts of the world and has become a global public health emergency. Later, on March 11, 2020, the World Health Organization declared this global outbreak as a pandemic. Two other highly pathogenic viruses of this family—SARS-CoV and Middle East respiratory syndrome coronavirus (MERS-CoV)—were also responsible for the earlier global outbreaks this century, with 11% and 34.4% case fatality rates (CFR), respectively.^2,3^ As of September 30, a total of 33,249,563 positive cases and 1,000,040 deaths have been confirmed worldwide.^4^ The symptoms of COVID-19–infected patients varies from mild to severe. The most common symptoms include fever, cough, muscle aches, fatigue, shortness of breath or trouble breathing, and a sore throat.^5^ The average incubation period for COVID-19 is 2 to 14 days; however, different incubation periods have been observed in a few places.^6,7^ Recent studies confirmed that respiratory droplets and close contact with infected patients are the major routes of human-to-human transmission.^8^

The highly contagious nature of SARS-CoV-2 and the lack of vaccines or effective treatments have made this pandemic an international crisis that affects the global healthcare system and triggers devastating social and economic consequences. Developed countries with modern healthcare facilities like France, Spain, Italy, and the USA have been affected massively. In response to the increasing number of COVID-19 cases and related deaths, many countries have implemented non-pharmaceutical physical interventions to halt the transmission of SARS-CoV-2, such as nationwide lockdowns, restriction to public movements, prohibiting public gatherings, ensuring physical distancing, closing educational institutes, canceling flights, and wearing face masks.^9^ The ultimate goal of such measurements is to reduce the infection rates by stopping the transmission chain, thereby buying some extra time for the arrangement or upgrading of existing healthcare infrastructure, training of the healthcare assistance, and enhancing testing facilities. Several countries, such as China, South Korea, Vietnam, and Singapore, reduced the case numbers dramatically through strict social distancing and mass testing measures.^10,11^ Among European countries, Italy first imposed nationwide lockdown measures. However, different European countries adopted different degrees of control measures to flatten the infection curves.

Similar to the developed countries, various low developed/developing countries resorted to comparable measures. To limit the spread of this new coronavirus, Bangladesh also acted to restrict the free movement of citizens since the identification of its first case on the March 8th, 2020. After confirming the first 33 COVID-19 patients, the government of Bangladesh (GoB) initially ordered a nationwide general holiday (similar to lockdown) from March 26th to April 9th, 2020, which included all educational institutions, commercial establishments, and shopping malls, and imposed restrictions on different transportation systems, including buses, trains, ships, and flights.^12^ Moreover, the GoB arranged the facilities of home and institutional quarantine for the exposed individuals/overseas travelers through the enhancement of contact tracing.^12^ The GoB also urged its citizens to strictly follow social distancing and maintain proper hygiene. The nationwide lockdown in Bangladesh was extended several times until May 30th. Unlike most other countries, Bangladesh also arranged emergency public health services and specially dedicated hospitals with COVID-19 units for isolating the affected individuals and ensuring proper treatment. Initially, polymerase chain reaction (PCR) based COVID-19 testing was only conducted by the Institute of Epidemiology, Disease Control and Research, Dhaka. Later, the testing facilities were gradually expanded nationwide to ensure sufficient testing opportunities for the suspected individuals. From June 1st, 2020 onwards, Bangladesh started to partly ease lockdown and restrictions on public movements were gradually relaxed.^13,14^

A few recent studies demonstrated that the human-to-human transmission of SARS-CoV-2 might be influenced by environmental factors like temperature, humidity, and wind speed.^15–17^ A group of researchers in China found that temperature significantly shapes the infection rates of SARS-CoV-2.^18^ The impact of temperature and humidity on the incidence and mortality due to the COVID-19 pandemic was investigated in ten European countries and this study found a significant correlation.^19^ A similar association has also been reported for Indonesia,^15^ Turkey,^20^ the Middle East Region,^17^ Singapore,^21^ Spain,^22^ the USA,^23^ and Norway.^24^ However, there is still considerable debate on the impact of meteorological parameters on COVID-19 cases, indicating that further scientific evidence is needed to clarify this relationship.

The current study aimed to identify the effectiveness of different administrative strategies against COVID-19 and analyze the impact of meteorological parameters, such as daily temperature, humidity, and wind speed, on the number of reported daily cases and mortality due to COVID-19 using statistical regression analysis. As the incubation period of COVID-19 varies from 1 to 14 days, we have evaluated the effect of each meteorological parameter at four time points, namely on day 0, 3, 7, and 14 before the respective clinical parameters.

## Results

### Impact of lockdown measures on the daily COVID-19 cases in Bangladesh

**Figure 1:**
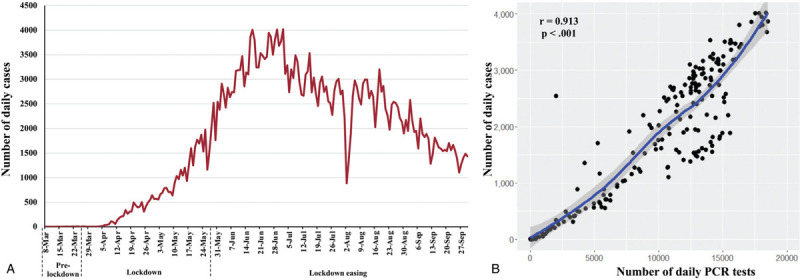
**The number of confirmed COVID-19 cases in Bangladesh from March 8th to September 30th, 2020**. A: The total number of daily cases were presented for 7 months and divided into three phases: pre-lockdown, lockdown, and lockdown easing phase. B: Regression analysis between the number of daily PCR tests and the number of daily confirmed cases. COVID-19: coronavirus disease 2019; PCR: polymerase chain reaction.

The first case of COVID-19 was reported in Bangladesh on March 8th, 2020, and we have analyzed the trend of daily cases until September 30th, 2020. The GoB imposed nationwide lockdown (general holiday) measures on the 26th of March, and followed-up with seven extensions until the 30th of May. To analyze the effectiveness of lockdown measures on the daily COVID-19 cases, we divided the entire period into three consecutive periods: pre-lockdown phase, lockdown phase, and lockdown easing phase. During the pre-lockdown phase from March 8th to March 25th, the total number of cases was only 44 and only 920 PCR tests were conducted (Figure 1A). This indicates that the actual number of cases might be higher than the reported cases.^25^ Importantly, over 663 thousand people entered the country between January 21st and March 26th.^25^ As a result of insufficient testing facilities and poor government monitoring, the number of daily cases stayed very low during the pre-lockdown phase.

During the lockdown phase, the number of daily COVID-19 cases increased gradually as the number of PCR tests and the testing facilities expanded across the country (Figure 1A). However, the daily case numbers increased dramatically in the latter part of the lockdown phase as thousands of people returned to the major cities after celebrating Eid-ul-Fitr (the biggest religious festival in Bangladesh), thereby ignoring the risks of infection and transmission. Therefore, the daily case numbers climbed rapidly to 2523 on May 29th. Despite the increasing number of new daily cases, the GoB eased the nationwide lockdown on May 31st. After reopening the daily activities (except educational institutes) with several health restrictions, daily cases escalated further and peaked at 4019 new cases on July 2nd (Figure 1A). The unwillingness of the citizens to follow the health instructions of social distancing amid the pandemic, wearing facial masks, and restrict movements are a few major reasons behind the sharp increase in the post-lockdown period. Several studies suggest that the robust implementation of control measures may result in the decline of the number of daily COVID-19 cases.^9,26–28^ The daily cases showed a gradual decline from the first week of July onwards (Figure 1A). Importantly, a direct positive correlation between the number of PCR tests and the number of daily new cases was observed (Figure 1B), indicating that reduced testing might have contributed to the observed decline in daily cases.

### Analysis of CFR, recovery rate, number of tests, and new cases

**Figure 2:**
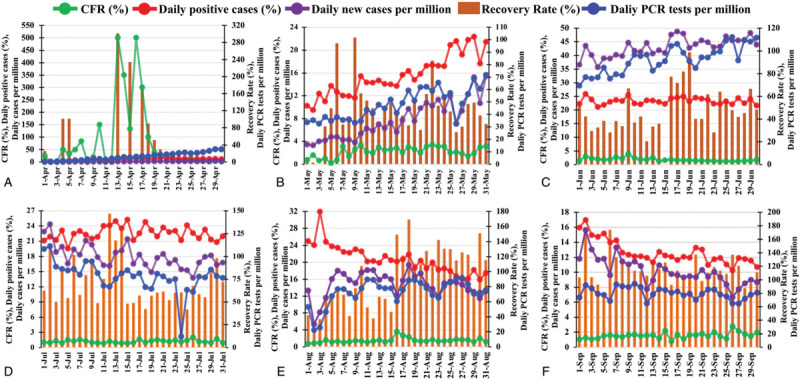
Analysis of COVID-19 case fatality rate, daily new cases, recovery rate, and daily PCR tests from April to September 2020 in Bangladesh. The COVID-19 case fatality rate (CFR), percentage of confirmed cases, total daily cases per 1 million and total daily PCR tests per 1 million are presented as line graphs and the COVID-19 recovery rate is presented as bar graphs for April (A), May (B), June (C), July (D), August (E), and September (F). COVID-19: coronavirus disease 2019; PCR: polymerase chain reaction.

Estimation of the CFR during an ongoing pandemic is critical due to the calculation biases and inconsistent data patterns. Many patients remained undiagnosed with COVID-19 during the initial outbreak phase because they were asymptomatic or due to the limited access of testing facilities, even for critically ill patients.^29^ Some factors are important and should be considered during the calculation process to minimize the biases, such as the number of tests, the average lag time between the onset of symptoms and deaths, healthcare facilities, coinfections, comorbidities, patient demographics, testing of both symptomatic and asymptomatic people, and the effectiveness of lockdown measures. Here, we have analyzed the month-wise data relative to the number of COVID-19 deaths, recovery, number of PCR tests, and percentage of positive cases from April to September, 2020. The CFR of COVID-19 in Bangladesh varies significantly due to the early biases, but after an initial spike in the first month, it decreased rapidly and remained at baseline level over the remaining five-month period (Figure 2). Initially, the average CFR for April was 81%, which was much higher than the CFR observed at a global level.^30,31^ There might be some critical elements behind this discrepancy, such as a lack of SARS-CoV-2 PCR testing, inadequately organized hospital beds and ICUs, personal protective equipment shortages for healthcare workers, and insufficient lockdown implementation.^32–34^ Moreover, testing was confined only to critically ill and hospitalized patients. Therefore, the number of confirmed COVID-19 related deaths was relatively higher. Over time, with the increasing number of PCR tests and adequate healthcare facilities, the CFR dropped dramatically with an average of 2.2%, 1.8%, 1.2%, 1.7%, and 1.6% for the months May, June, July, August, and September, respectively. Although there was a slight increase over the last 2 months, the average CFR per month remained steady with the enhancement of other medical and administrative measures.

Three critical elements that might influence the CFR largely are: (a) the rate of recovery of the PCR confirmed patients, (b) the number of PCR tests performed per million population, and (c) the total number of new positive cases per million/percentage of the daily positive cases for each month. Since all these parameters have a large impact on the previously explained CFR (Figure 2), we have further analyzed these in more detail. Most COVID-19 positive people show mild to medium illness,^35^ but when patients do not take sufficient rest and medication, manageable symptoms might turn into life-threatening conditions that could ultimately result in death.^36^ Thus, we have tracked the infection rate alongside the recovery rate. But we were only able to analyze the data of confirmed cases, as the data for patients showing only COVID-19 symptoms without performing tests were not available. However, a steady increase in the overall COVID-19 recovery rate was observed starting from 40% in April to more than 100% in August and September (Figure 2). Similarly, the number of PCR tests per million showed a gradual enhancement with values of 0.85, 30.07, 71.94, and 111.61 at the end of March, April, May, and June, respectively. However, due to a religious festival and natural adversities, the number of PCR tests per million went down to 76.41, 75.444, and 81.19 at the end of July, August, and September, respectively. Interestingly, the number of new positive cases and the percentage of positive tested cases showed a similar upwards trend along with the number of daily PCR tests in the first three months (April–June). However, a declining trend was observed over the last 3 months (July–September), where the number of positive cases decreased down to only 10% on the 30th of September.

### Correlation between meteorological parameters and COVID-19 daily cases and deaths

**Figure 3:**
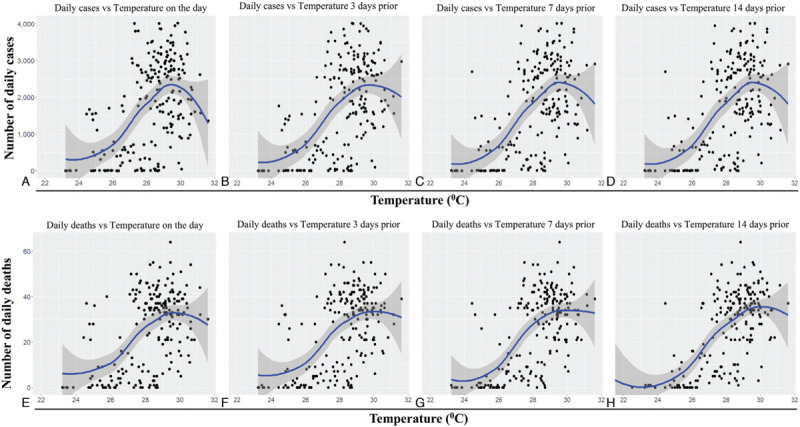
**Trend of daily mean temperature versus daily new cases and deaths due to the COVID-19 pandemic in Bangladesh**. The graphs represent scatterplots of daily new COVID-19 cases (A–D) or daily COVID-19 deaths (E–H) versus the daily mean temperature on the day (A and E), 3 days prior (B and F), 7 days prior (C and G) and 14 days prior (D and H). The blue lines represent regression analysis with confidence intervals depicted in shaded dark grey. COVID-19: coronavirus disease 2019.

Pearson correlation tests were performed to examine the correlation between the number of daily confirmed cases and deaths with the meteorological variables daily mean temperature, humidity, and wind speed. These meteorological parameters were considered as independent variables, while the clinical data of four time points, namely on the day, and after 3, 7, and 14 days were considered as dependent variables. The correlation between temperature and daily confirmed cases was positive for all four time points (Table 1, Figure 3A–D). Interestingly, a negative correlation trend appears to become visible upwards of 29.5°C. Importantly, the strongest correlation was observed for the daily positive COVID-19 cases with the temperature 14 days prior (*r* = 0.620, *P* < 0.001; Table 1). Similar correlations were observed between the average temperature and the daily COVID-19 deaths (Table 1, Figure 3E–H). Again, the strongest correlation was observed between the daily deaths and the temperature 14 days prior (*r* = 0.661, *P* < 0.001; Table 1).

**Table 1:** The overall correlation coefficient between the daily new cases and deaths due to COVID-19 pandemic with the daily mean temperature, humidity, and wind speed in Bangladesh from April to September 2020

Parameters	Correlation coefficient, *r*	95% CI	*R* ^2^	*β*	Standard error	Significance
Temperature on the day versus daily cases	0.486	0.347–0.584	0.236	345.150	43.345	*P *< 0.001
Temperature 3 days prior versus daily cases	0.536	0.431–0.627	0.287	369.916	40.680	*P* < 0.001
Temperature 7 days prior versus daily cases	0.560	0.459–0.647	0.314	385.221	39.745	*P* < 0.001
Temperature 14 days prior versus daily cases	0.620	0.528–0.697	0.384	396.898	35.087	*P* < 0.001
Temperature on the day versus daily deaths	0.495	0.384–0.591	0.245	4.838	0.593	*P* < 0.001
Temperature 3 days prior versus daily deaths	0.538	0.434–0.628	0.291	5.114	0.560	*P* < 0.001
Temperature 7 days prior versus daily deaths	0.596	0.500–0.677	0.261	2.583	0.314	*P* < 0.001
Temperature 14 days prior versus daily deaths	0.661	0.577–0.731	0.437	5.829	0.462	*P* < 0.001
Humidity on the day versus daily cases	0.611	0.518–0.690	0.374	60.289	5.451	*P *< 0.001
Humidity 3 days prior versus daily cases	0.624	0.533–0.716	0.390	61.428	5.367	*P *< 0.001
Humidity 7 days prior versus daily cases	0.643	0.555–0.716	0.413	62.355	5.192	*P *< 0.001
Humidity 14 days prior versus daily cases	0.653	0.567–0.725	0.427	62.102	5.028	*P *< 0.001
Humidity on the day versus daily deaths	0.635	0.547–0.711	0.404	0.863	0.073	*P *< 0.001
Humidity 3 days prior versus daily deaths	0.636	0.547–0.711	0.404	0.861	0.073	*P *< 0.001
Humidity 7 days prior versus daily deaths	0.644	0.556–0.717	0.414	0.860	0.071	*P *< 0.001
Humidity 14 days prior versus daily deaths	0.673	0.591–0.741	0.453	0.881	0.068	*P *< 0.001
Wind Speed on the day versus daily cases	0.262	0.130–0.384	0.068	158.52	40.819	*P *< 0.001
Wind Speed 3 days prior versus daily cases	0.2	0.065–0.327	0.04	118.556	40.529	*P *< 0.001
Wind Speed 7 days prior versus daily cases	0.311	0.183–0.429	0.097	187.514	39.985	*P *< 0.001
Wind Speed 14 days prior versus daily cases	0.329	0.212–0.446	0.109	197.876	39.590	*P *< 0.001
Wind speed on the day versus daily deaths	0.172	0.036–0.301	0.029	1.431	0.574	*P *< 0.013
Wind speed 3 days prior versus daily deaths	0.119	-0.02–0.251	0.014	0.969	0.565	*P *< 0.088
Wind speed 7 days prior versus daily deaths	0.275	0.144–0.397	0.076	2.285	0.557	*P *< 0.001
Wind speed 14 days prior versus daily deaths	0.352	0.226–0.466	0.124	2.908	0.540	*P *< 0.001

CI: confidence interval; COVID-19: coronavirus disease 2019.

**Figure 4:**
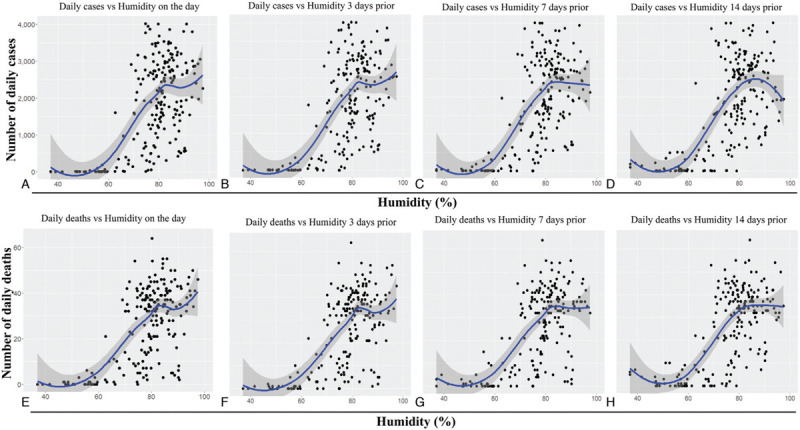
**Trend of daily mean humidity versus daily new cases and deaths due to the COVID-19 pandemic in Bangladesh**. The graphs represent scatterplots of daily new COVID-19 cases (A–D) or daily COVID-19 deaths (E–H) versus the daily mean humidity on the day (A and E), 3 days prior (B and F), 7 days prior (C and G) and 14 days prior (D and H). The blue lines represent regression analysis with confidence intervals depicted in shaded dark grey. COVID-19: coronavirus disease 2019.

**Figure 5:**
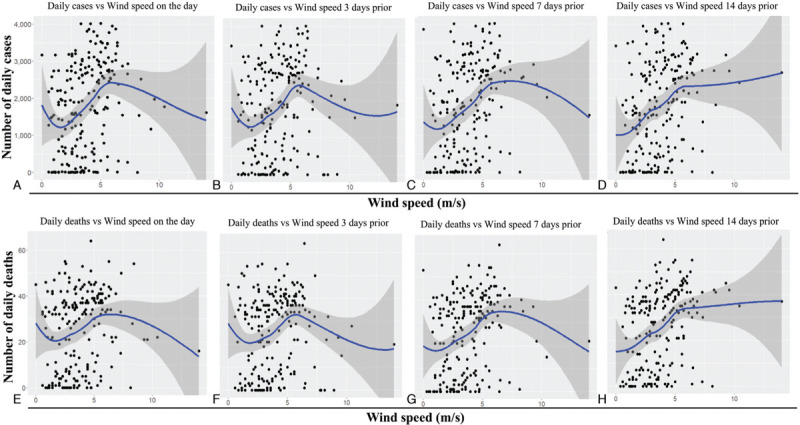
**Trend of daily mean wind speed versus daily new cases and deaths due to the COVID-19 pandemic in Bangladesh**. The graphs represent scatterplots of daily new COVID-19 cases (A–D) or daily COVID-19 deaths (E–H) versus the daily mean wind speed on the day (A and E), 3 days prior (B and F), 7 days prior (C and G) and 14 days prior (D and H). The blue lines represent regression analysis with confidence intervals depicted in shaded dark grey. COVID-19: coronavirus disease 2019.

Similarly, a significant positive correlation was observed between daily mean humidity, and the daily confirmed cases and daily deaths (Table 1 and Figure 4), indicating that an increase in humidity results in higher daily cases and deaths. Similar to what was observed for the average temperature, the average humidity 14 days prior showed the strongest correlation with daily cases (*r* = 0.653, *P* < 0.001; Table 1) and daily deaths (*r* = 0.673, *P* < 0.001; Table 1). Finally, we evaluated the correlation between the daily wind speed and the number of COVID-19 cases and associated deaths (Table 1, Figure 5). Although the correlation was not as strong as observed for temperature and humidity, a positive correlation was still observed, particularly for the wind speed 14 days prior and the daily new cases (*r* = 0.329, *P* < 0.001; Table 1) and the daily deaths (*r* = 0.352, *P* < 0.001; Table 1). Overall, these results imply that daily confirmed cases and deaths are positively correlated with temperature, humidity and wind speed, most strongly with the meteorological parameters observed 14 days prior, which is likely related with the incubation period of COVID-19.

### Regression analysis between meteorological parameters and daily cases and deaths

Regression analyses were performed to determine the changes in daily confirmed COVID-19 cases and deaths with a 1-unit change in meteorological parameters. Regression analysis between temperature and daily cases and deaths showed that with an increase of 1°C 14 days prior, the number of daily cases and deaths increased by 396.898 (*P* < 0.001) and 5.829 (*P* < 0.001), respectively (Table 1). Similarly, for every 1% increase in humidity 14 days prior, the daily new cases of COVID-19 increased by 62.102 (*P* < 0.001) and daily deaths increased by 0.881 (*P* < 0.001), while for every m/s increase in wind speed 14 days prior the daily cases and daily deaths increased by 197.876 (*P* < 0.001) and 2.908 (*P* < 0.001), respectively.

## Discussion

The distressing situation caused by the COVID-19 pandemic resulted in a global crisis with devastating consequences for our daily life. In this study, we have explored the impact of lockdown and meteorological parameters on COVID-19 confirmed daily cases and deaths in Bangladesh. The data from April to September 2020 were analyzed. Previous studies have shown that strict lockdown measures were effective in combating the spread of COVID-19 cases in several countries, including China, South Korea, Singapore, and Spain.^11,26,27,37,38^ Like many countries, Bangladesh also enforced a nationwide lockdown as non-pharmaceutical intervention to interrupt the spread of the COVID-19. A recent study has suggested that robust implementation of lockdown and strict social distancing can results in a 60% reduction of transmission of SARS-CoV-2, considering both symptomatic and asymptomatic patients.^39^ However, in most of the countries, including Bangladesh, lockdown did not display immediate effects. Since the beginning of the pandemic, thousands of people from all around the world have entered the country without proper scanning, testing, quarantine, and isolation. Bangladesh is also densely populated, with a large portion of the population depending on daily earnings. As a result, due to inadequate systemic testing and inefficient implementations of the lockdown, the actual impact on daily cases is poorly understood.

The transmission of respiratory virus infections, including SARS-CoV-2, is complex and depends on many factors. The virus can persist on contaminated environmental surfaces and is affected by environmental conditions. Several studies have suggested that temperature, humidity, and wind speed might influence the survival and spread of SARS-CoV-2.^17,40^ A positive linear relationship has been reported between the mean temperature and the number of COVID-19 cases in China.^41^ The authors identified that below 3°C mean temperature, a 1°C increase in temperature was associated with a 4.861% increase in the case numbers. In contrast, a negative impact of temperature and humidity was found in a recent report.^42^ These contrasting findings might be defined by diverse geographical locations and weather conditions. Meo et al.^19^ also observed a significant positive correlation between temperature and daily cases and deaths in several European countries. A positive correlation between wind speed and daily cases was also reported for Turkey.^20^ The authors identified that the wind speed 14 days prior had the highest positive correlation with the daily cases (*r* = 0.550). Our study also found a similar positive association between wind speed, daily cases, and daily deaths.

Several studies also suggested a positive association between environmental factors and transmissibility of coronaviruses like SARS-CoV and Middle East respiratory syndrome coronavirus.^43–45^ In the present study, we identified that both daily cases and deaths were increased with an increase in temperature, humidity, and wind speed. Similar results were found in Spain, where the number of daily cases and daily deaths increased significantly with a 1% increase in humidity by 34.27% and 3.998%, respectively.^19^

## Conclusions

This study investigated the impact of lockdown measures and different meteorological indicators, such as mean temperature, humidity, and wind speed, on COVID-19 confirmed cases and deaths using data from Bangladesh. Our results showed that all meteorological parameters were significantly and positively correlated with the number of confirmed cases and deaths. Based on regression analysis we concluded that an increase in temperature, humidity, and wind speed resulted in increased transmission of COVID-19 in Bangladesh. These findings might provide useful information for governments and authorities and insights into the dependency of COVID-19 on meteorological parameters. We strongly believe that essential steps should be taken to prevent transmission based on rational studies. However, the present study has some limitations; further study is recommended considering the regional variations in Bangladesh.

## Materials and methods

### Data collection

COVID-19 case-related data from March 08, 2020 (first reported case in Bangladesh) to September 30, 2020, were collected from the official website of the Directorate General of Health Services, Ministry of Health and Family Welfare, Government of People's Republic of Bangladesh (https://corona.gov.bd/press-release). The mean epidemiological and weather data of Bangladesh, such as daily temperature, were obtained from ten different meteorological stations from the National Oceanic and Atmospheric Administration Center (https://www.noaa.gov/weather) and then the average temperature was calculated. Daily information of humidity and wind speed was recorded from the climate web “Time and Date” (https://www.timeanddate.com/) and the mean daily value was calculated by averaging the hourly data. These environmental data were analyzed in relation to the COVID-19 cases and morality numbers on the day of the cases or deaths or 3, 7, and 14 days prior.

### Calculation of CFR and recovery rate

The CFR is commonly used to determine the potential threat or severity of any disease and is defined as the number of deaths relative to the number of total identified cases. However, during an ongoing epidemic, the situation might be different as the patient who died might be infected earlier and the full denominator remains unknown because of asymptomatic cases. By considering the average incubation period of 14 days estimated between the first symptoms to death, we calculated the CFR by dividing the number of deaths on a specific day by the total number of confirmed cases 14 days prior.^30,46^ Though the study period was from March 8th to September 30th, 2020, the CFR was calculated from April onwards as the data was either not available or to inconsistent to calculate the CFR. The recovery rate was also calculated using a 14 days incubation period and was defined as the number of patients recovered on a specific day divided by the total number of confirmed cases 14 days prior.

### Statistical regression analysis

Pearson correlation (*r*) tests were performed to analyze the correlation between daily mean temperature, humidity, and wind speed with the number of daily COVID-19 cases and its associated deaths. The number of daily confirmed cases and daily deaths were considered as the dependent variables, while the three meteorological parameters were used as independent variables. Linear regression analysis was used to model the relationship between meteorological parameters and the number of daily cases and daily deaths. The R software package (version 4.0.2) and Microsoft Excel (version 16.0.13231.20372) were used to perform the statistical analyses. All the statistical analyses were two-sided and a value *P* < 0.05 was considered as statistically significant. Supplementary Digital Content 1.

## Supplementary Material

Supplemental Digital Content
