# Supplementary material for: Impact of Lockdown Measures and Meteorological Parameters on the COVID-19 Incidence and Mortality Rate in Bangladesh
Source: Infect Microbes Dis. 2021 Feb 5;3(1):41–8. doi: 10.1097/IM9.0000000000000052 (PMC8011345; doi:10.1097/IM9.0000000000000052)
Supplement: Supplemental Digital Content [file im9-3-41-s001.docx]

**Supplementary Digital Content 1 Detailed data of various parameters associated with the COVID-19 pandemic from April to September, 2020, in Bangladesh**

| **Date** | **Case fatality rate** | **Recovery rate** | **Percentage of positive cases** | **Daily PCR test per million** | **Daily cases per million** |
| --- | --- | --- | --- | --- | --- |
| 1-Apr | 25 | 25 | 1.91 | 0.95 | 0.02 |
| 2-Apr | 0 | 0 | 1.42 | 0.85 | 0.01 |
| 3-Apr | 0 | 0 | 2.46 | 1.23 | 0.03 |
| 4-Apr | 50 | 100 | 2.07 | 2.63 | 0.05 |
| 5-Apr | 33.333 | 100 | 4.90 | 2.22 | 0.11 |
| 6-Apr | 50 | 0 | 7.48 | 2.83 | 0.21 |
| 7-Apr | 83.333 | 0 | 6.04 | 4.11 | 0.25 |
| 8-Apr | NA | NA | 5.50 | 5.94 | 0.33 |
| 9-Apr | 20 | 0 | 12.38 | 5.48 | 0.68 |
| 10-Apr | 150 | 0 | 7.94 | 7.17 | 0.57 |
| 11-Apr | NA | NA | 6.05 | 5.80 | 0.35 |
| 12-Apr | NA | NA | 10.37 | 8.12 | 0.84 |
| 13-Apr | 500 | 300 | 11.59 | 9.51 | 1.10 |
| 14-Apr | 350 | 0 | 10.97 | 11.54 | 1.27 |
| 15-Apr | 133.33 | 233.33 | 12.59 | 10.54 | 1.33 |
| 16-Apr | 500 | 0 | 16.89 | 12.23 | 2.07 |
| 17-Apr | 300 | 180 | 12.15 | 13.27 | 1.61 |
| 18-Apr | 100 | 88.89 | 14.47 | 12.80 | 1.85 |
| 19-Apr | 38.89 | 50.00 | 11.85 | 15.95 | 1.89 |
| 20-Apr | 28.57 | 28.57 | 17.70 | 16.83 | 2.98 |
| 21-Apr | 21.95 | 4.88 | 14.59 | 18.01 | 2.63 |
| 22-Apr | 18.52 | 9.26 | 12.60 | 18.75 | 2.36 |
| 23-Apr | 6.25 | 14.29 | 12.12 | 20.69 | 2.51 |
| 24-Apr | 4.26 | 4.26 | 13.65 | 22.33 | 3.05 |
| 25-Apr | 15.52 | 1.72 | 9.26 | 20.21 | 1.87 |
| 26-Apr | 3.60 | 6.47 | 12.03 | 21.05 | 2.53 |
| 27-Apr | 3.85 | 4.95 | 13.04 | 23.09 | 3.01 |
| 28-Apr | 1.44 | 3.83 | 12.67 | 26.24 | 3.33 |
| 29-Apr | 3.65 | 5.02 | 12.90 | 30.09 | 3.88 |
| 30-Apr | 1.76 | 2.93 | 11.36 | 30.07 | 3.42 |
| 1-May | 0.75 | 5.26 | 10.25 | 33.76 | 3.46 |
| 2-May | 1.63 | 0.98 | 9.47 | 35.30 | 3.34 |
| 3-May | 0.64 | NA | 12.39 | 32.51 | 4.03 |
| 4-May | 1.02 | 29.88 | 10.99 | 37.92 | 4.17 |
| 5-May | 0.23 | 44.47 | 13.76 | 34.59 | 4.76 |
| 6-May | 0.77 | 96.67 | 12.66 | 37.80 | 4.79 |
| 7-May | 3.14 | 31.40 | 12.03 | 35.54 | 4.28 |
| 8-May | 1.39 | 37.97 | 11.93 | 35.99 | 4.29 |
| 9-May | 2.59 | 101.29 | 11.64 | 33.10 | 3.85 |
| 10-May | 3.35 | 56.46 | 15.46 | 34.76 | 5.37 |
| 11-May | 2.21 | 50.70 | 14.35 | 43.66 | 6.26 |
| 12-May | 2.00 | 44.63 | 14.31 | 41.03 | 5.87 |
| 13-May | 2.96 | 33.39 | 14.71 | 47.85 | 7.04 |
| 14-May | 2.48 | 42.91 | 14.08 | 44.77 | 6.31 |
| 15-May | 2.63 | 48.86 | 14.01 | 51.98 | 7.28 |
| 16-May | 2.90 | 42.57 | 13.71 | 41.08 | 5.63 |
| 17-May | 2.11 | 38.50 | 15.69 | 49.15 | 7.71 |
| 18-May | 3.05 | 30.81 | 16.37 | 59.29 | 9.70 |
| 19-May | 2.67 | 51.91 | 14.81 | 51.18 | 7.58 |
| 20-May | 2.03 | 27.09 | 15.84 | 61.83 | 9.79 |
| 21-May | 3.12 | 55.95 | 17.28 | 62.16 | 10.74 |
| 22-May | 3.39 | 82.93 | 17.42 | 58.92 | 10.26 |
| 23-May | 3.14 | 46.54 | 17.29 | 65.62 | 11.35 |
| 24-May | 3.16 | 55.81 | 17.20 | 53.96 | 9.28 |
| 25-May | 2.03 | 41.88 | 20.90 | 57.25 | 11.96 |
| 26-May | 2.17 | 25.28 | 21.56 | 32.75 | 7.06 |
| 27-May | 1.89 | 29.78 | 19.23 | 48.55 | 9.33 |
| 28-May | 1.44 | 48.03 | 21.79 | 56.39 | 12.29 |
| 29-May | 1.91 | 49.08 | 22.33 | 68.45 | 15.28 |
| 30-May | 3.01 | 38.71 | 17.66 | 60.49 | 10.68 |
| 31-May | 3.14 | 31.89 | 21.43 | 71.94 | 15.42 |
| 1-Jun | 1.37 | 50.94 | 20.81 | 69.29 | 14.42 |
| 2-Jun | 2.96 | 41.81 | 22.91 | 76.95 | 17.63 |
| 3-Jun | 2.29 | 29.07 | 21.54 | 75.78 | 16.32 |
| 4-Jun | 1.97 | 32.21 | 19.09 | 76.89 | 14.68 |
| 5-Jun | 1.77 | 37.96 | 20.07 | 85.33 | 17.13 |
| 6-Jun | 1.87 | 27.82 | 21.10 | 75.63 | 15.96 |
| 7-Jun | 2.74 | 37.73 | 20.88 | 79.57 | 16.61 |
| 8-Jun | 2.13 | 33.27 | 21.13 | 78.40 | 16.57 |
| 9-Jun | 3.86 | 66.55 | 21.62 | 88.82 | 19.21 |
| 10-Jun | 2.40 | 36.53 | 19.98 | 96.70 | 19.32 |
| 11-Jun | 1.82 | 41.79 | 20.21 | 95.53 | 19.30 |
| 12-Jun | 1.82 | 19.90 | 21.71 | 96.85 | 21.02 |
| 13-Jun | 2.49 | 32.77 | 20.94 | 82.61 | 17.30 |
| 14-Jun | 1.26 | 35.48 | 21.65 | 87.86 | 19.03 |
| 15-Jun | 1.60 |  | 20.61 | 91.09 | 18.77 |
| 16-Jun | 1.82 | 76.85 | 22.44 | 104.27 | 23.39 |
| 17-Jun | 1.60 | 71.43 | 22.87 | 106.16 | 24.28 |
| 18-Jun | 1.57 | 81.51 | 23.39 | 98.48 | 23.04 |
| 19-Jun | 1.59 | 98.34 | 21.56 | 91.13 | 19.64 |
| 20-Jun | 1.40 | 39.77 | 23.09 | 84.99 | 19.63 |
| 21-Jun | 1.42 | 39.52 | 22.66 | 94.40 | 21.39 |
| 22-Jun | 1.39 | 61.35 | 22.37 | 94.22 | 21.08 |
| 23-Jun | 1.36 | 27.75 | 20.94 | 98.68 | 20.67 |
| 24-Jun | 1.16 | 63.67 | 21.07 | 99.54 | 20.97 |
| 25-Jun | 1.22 | 57.39 | 21.92 | 109.02 | 23.90 |
| 26-Jun | 1.15 | 47.19 | 20.91 | 112.05 | 23.43 |
| 27-Jun | 1.19 | 41.49 | 23.12 | 91.81 | 21.22 |
| 28-Jun | 1.37 | 44.86 | 21.05 | 109.63 | 23.07 |
| 29-Jun | 1.45 | 66.25 | 22.50 | 108.04 | 24.31 |
| 30-Jun | 1.66 | 47.80 | 19.98 | 111.61 | 22.30 |
| 1-Jul | 1.02 | 61.98 | 21.12 | 108.27 | 22.87 |
| 2-Jul | 1.00 | 113.96 | 21.89 | 111.22 | 24.34 |
| 3-Jul | 1.30 | 49.52 | 21.26 | 88.74 | 18.86 |
| 4-Jul | 0.90 | 82.50 | 23.11 | 86.18 | 19.92 |
| 5-Jul | 1.56 | 53.92 | 19.57 | 84.73 | 16.58 |
| 6-Jul | 1.26 | 101.26 | 22.47 | 86.28 | 19.39 |
| 7-Jul | 1.61 | 57.24 | 22.98 | 79.79 | 18.34 |
| 8-Jul | 1.33 | 79.03 | 22.26 | 94.93 | 21.13 |
| 9-Jul | 1.04 | 93.92 | 21.49 | 94.69 | 20.35 |
| 10-Jul | 0.96 | 48.14 | 21.86 | 81.70 | 17.86 |
| 11-Jul | 0.86 | 75.00 | 24.00 | 67.80 | 16.27 |
| 12-Jul | 1.23 | 146.50 | 24.11 | 66.99 | 16.15 |
| 13-Jul | 0.97 | 117.16 | 24.95 | 75.25 | 18.77 |
| 14-Jul | 0.90 | 133.35 | 23.51 | 81.49 | 19.16 |
| 15-Jul | 0.87 | 47.58 | 25.23 | 84.81 | 21.40 |
| 16-Jul | 0.97 | 48.49 | 21.20 | 78.07 | 16.55 |
| 17-Jul | 1.64 | 56.58 | 22.54 | 81.53 | 18.38 |
| 18-Jul | 1.03 | 41.76 | 24.80 | 66.16 | 16.41 |
| 19-Jul | 1.35 | 56.39 | 23.14 | 64.36 | 14.89 |
| 20-Jul | 1.56 | 59.79 | 21.91 | 80.94 | 17.74 |
| 21-Jul | 1.35 | 60.82 | 23.70 | 78.13 | 18.52 |
| 22-Jul | 1.20 | 51.73 | 22.77 | 72.99 | 16.62 |
| 23-Jul | 1.49 | 59.70 | 23.04 | 75.10 | 17.30 |
| 24-Jul | 1.19 | 59.95 | 21.19 | 12.28 | 15.43 |
| 25-Jul | 1.41 | 41.47 | 24.12 | 63.27 | 15.26 |
| 26-Jul | 2.03 | 67.22 | 22.57 | 61.04 | 13.78 |
| 27-Jul | 1.19 | 58.12 | 21.56 | 77.89 | 16.79 |
| 28-Jul | 1.11 | 54.73 | 23.28 | 77.01 | 17.93 |
| 29-Jul | 0.99 | 81.46 | 21.30 | 85.57 | 18.23 |
| 30-Jul | 1.76 | 97.62 | 20.83 | 78.36 | 16.32 |
| 31-Jul | 0.92 | 71.72 | 21.98 | 76.41 | 16.79 |
| 1-Aug | 0.78 | 41.23 | 24.98 | 53.32 | 13.32 |
| 2-Aug | 0.89 | 23.83 | 24.05 | 22.31 | 5.37 |
| 3-Aug | 1.02 | 36.41 | 31.91 | 25.74 | 8.21 |
| 4-Aug | 1.64 | 63.95 | 24.87 | 46.71 | 11.62 |
| 5-Aug | 1.20 | 68.88 | 23.78 | 67.60 | 16.08 |
| 6-Aug | 1.37 | 72.62 | 23.43 | 76.97 | 18.03 |
| 7-Aug | 1.06 | 69.07 | 22.45 | 76.92 | 17.27 |
| 8-Aug | 1.27 | 40.48 | 22.25 | 71.09 | 15.82 |
| 9-Aug | 1.49 | 77.63 | 23.12 | 65.17 | 15.06 |
| 10-Aug | 1.41 | 107.03 | 22.62 | 77.83 | 17.61 |
| 11-Aug | 1.11 | 51.86 | 20.22 | 89.77 | 18.15 |
| 12-Aug | 1.40 | 37.12 | 20.30 | 89.35 | 18.14 |
| 13-Aug | 1.63 | 66.12 | 19.88 | 79.72 | 15.85 |
| 14-Aug | 1.23 | 63.20 | 21.52 | 77.87 | 16.75 |
| 15-Aug | 1.55 | 46.02 | 20.51 | 78.08 | 16.02 |
| 16-Aug | 3.61 | 148.42 | 20.20 | 60.68 | 12.26 |
| 17-Aug | 2.73 | 121.02 | 20.72 | 75.85 | 15.72 |
| 18-Aug | 2.40 | 168.61 | 21.87 | 88.62 | 19.38 |
| 19-Aug | 1.54 | 109.76 | 18.72 | 88.91 | 16.64 |
| 20-Aug | 1.38 | 109.27 | 20.40 | 85.16 | 17.37 |
| 21-Aug | 1.37 | 127.11 | 18.55 | 78.40 | 14.54 |
| 22-Aug | 1.76 | 113.06 | 19.95 | 68.79 | 13.72 |
| 23-Aug | 1.37 | 141.70 | 18.27 | 65.42 | 11.95 |
| 24-Aug | 1.44 | 130.17 | 18.57 | 81.06 | 15.05 |
| 25-Aug | 1.50 | 129.54 | 17.98 | 85.73 | 15.42 |
| 26-Aug | 1.80 | 114.42 | 16.72 | 91.28 | 15.26 |
| 27-Aug | 1.72 | 125.14 | 16.11 | 91.61 | 14.76 |
| 28-Aug | 1.70 | 122.13 | 16.09 | 83.23 | 13.39 |
| 29-Aug | 1.21 | 76.66 | 18.23 | 70.80 | 12.91 |
| 30-Aug | 2.08 | 150.40 | 15.90 | 72.29 | 11.49 |
| 31-Aug | 1.27 | 114.84 | 17.46 | 75.44 | 13.17 |
| 1-Sep | 1.09 | 102.81 | 15.97 | 73.95 | 11.81 |
| 2-Sep | 1.27 | 176.16 | 16.98 | 92.09 | 15.64 |
| 3-Sep | 1.12 | 103.35 | 14.96 | 87.36 | 13.07 |
| 4-Sep | 1.21 | 92.09 | 14.76 | 79.19 | 11.68 |
| 5-Sep | 1.55 | 73.33 | 15.18 | 77.82 | 11.81 |
| 6-Sep | 1.62 | 173.49 | 14.02 | 68.77 | 9.64 |
| 7-Sep | 1.49 | 132.72 | 14.29 | 93.35 | 13.34 |
| 8-Sep | 1.41 | 127.15 | 12.64 | 90.69 | 11.46 |
| 9-Sep | 1.63 | 118.90 | 12.38 | 89.37 | 11.07 |
| 10-Sep | 1.68 | 112.73 | 12.16 | 94.24 | 11.46 |
| 11-Sep | 1.54 | 111.90 | 12.15 | 89.33 | 10.85 |
| 12-Sep | 1.60 | 105.44 | 11.96 | 64.95 | 7.77 |
| 13-Sep | 1.63 | 125.04 | 11.35 | 78.74 | 8.94 |
| 14-Sep | 1.20 | 115.55 | 12.75 | 86.11 | 10.98 |
| 15-Sep | 2.21 | 125.08 | 12.27 | 85.10 | 10.44 |
| 16-Sep | 0.81 | 91.98 | 12.09 | 80.92 | 9.78 |
| 17-Sep | 1.67 | 113.21 | 11.65 | 82.82 | 9.65 |
| 18-Sep | 1.14 | 99.69 | 12.11 | 77.11 | 9.33 |
| 19-Sep | 1.64 | 105.18 | 11.95 | 79.41 | 9.49 |
| 20-Sep | 1.63 | 136.87 | 13.32 | 70.21 | 9.35 |
| 21-Sep | 1.82 | 97.73 | 13.06 | 79.06 | 10.33 |
| 22-Sep | 1.48 | 109.57 | 10.99 | 85.79 | 9.43 |
| 23-Sep | 2.03 | 118.39 | 11.77 | 85.71 | 10.09 |
| 24-Sep | 1.48 | 113.05 | 11.94 | 78.14 | 9.33 |
| 25-Sep | 1.17 | 107.81 | 11.09 | 75.55 | 8.38 |
| 26-Sep | 2.81 | 136.74 | 10.27 | 65.21 | 6.70 |
| 27-Sep | 2.17 | 120.19 | 11.93 | 64.72 | 7.72 |
| 28-Sep | 1.77 | 87.31 | 11.80 | 72.21 | 8.52 |
| 29-Sep | 1.51 | 94.26 | 11.56 | 77.95 | 9.01 |
| 30-Sep | 1.98 | 110.77 | 10.71 | 81.19 | 8.70 |
